# Temporal changes of microvascular obstruction and infarct border zone after acute myocardial infarction assessed by contrast enhanced magnetic resonance imaging

**DOI:** 10.1186/1532-429X-13-S1-P106

**Published:** 2011-02-02

**Authors:** Birgit Langhans, Tareq Ibrahim, Markus Schwaiger, Albert Schömig, Martin Hadamitzky

**Affiliations:** 1 Deutsches Herzzentrum München, Munich, Germany; 2 Klinikum Rechts der Isar/ TUM, Munich, Germany

## Background

Besides total scar size, scar characterization parameters are emerging as additional prognostic factors after acute myocardial infarction. Of these, microvascular obstruction (MVO) and infarct border zone are the most promising ones. Nevertheless, a MRI scan performed few days after infarction assesses a rapidly changing myocardium. In particular the infarct border zone that is believed to be a substrate for malignant arrhythmias, may change considerably during subsequent remodeling.

## Methods

We analyzed 165 patients with AMI and primary angioplasty who underwent contrast enhanced MRI imaging both early (4 days in median) and late (6 months in median) after the acute event. MRI scar size was measured 15 minutes after gadolinium injection. Infarct border zone was defined as area with signal intensity between 3 and 6 standard deviations above healthy myocardium, infarct core as signal intensity above 6 standard deviations above healthy myocardium.

## Results

Four days after infarction, MVO was present in 70 patients (42%) with a median size of 2.1 ml (IQR 0.9-5.2ml). After 6 months MVO was still present in 10 patients (6%) with a median size of 0.7 ml (IQR 0.5-1.6 ml). Infarct border zone was significantly smaller after 6 months (median 7.2 ml, IQR 4.0-10.2ml), when compared with the scan after 4 days (median 8.9 ml, IQR 5.9-14.9ml, p<0.001), while the size of infarct core zone did not change significantly (median 7.4 ml after 6 month, IQR 2.9-15.0, compared to 6.9 ml after 4 days, IQR 2.5-15.9, p=0.33).

## Conclusions

MVO is still present after 6 months in a small fraction of patients. Contrary to the infarct core zone, the infarct border zone shrinks significantly during remodeling after acute myocardial infarction. Whether these changes have prognostic implications has to be tested on a larger patient population.

